# Organ Dysfunction among Piglets Treated with Inhaled Nitric Oxide and Intravenous Hydrocortisone during Prolonged Endotoxin Infusion

**DOI:** 10.1371/journal.pone.0096594

**Published:** 2014-05-14

**Authors:** Sofie Paues Göranson, Waldemar Goździk, Piotr Harbut, Stanisław Ryniak, Stanisław Zielinski, Caroline Gillis Haegerstrand, Andrzej Kübler, Göran Hedenstierna, Claes Frostell, Johanna Albert

**Affiliations:** 1 Department of Anesthesia and Intensive Care, Karolinska Institutet, Danderyd Hospital, Stockholm, Sweden; 2 Department of Anesthesiology and Intensive Therapy, Wroclaw University of Medicine, Wroclaw, Poland; 3 Department of Medical Sciences, Uppsala University Hospital, Uppsala, Sweden; Biological Research Centre of the Hungarian Academy of Sciences, Hungary

## Abstract

**Objective:**

It has previously been shown that a combination of inhaled nitric oxide (iNO) and intravenous (IV) steroid attenuates endotoxin-induced organ damage in a 6-hour porcine endotoxemia model. We aimed to further explore these effects in a 30-hour model with attention to clinically important variables.

**Design:**

Randomized controlled trial.

**Setting:**

University animal laboratory.

**Subjects:**

Domestic piglets (n = 30).

**Interventions:**

Animals were randomized into 5 groups (n = 6 each): 1) Controls, 2) LPS-only (endotoxin/lipopolysaccharide (LPS) infusion), 3) LPS + iNO, 4) LPS + IV steroid, 5) LPS + iNO + IV steroid.

**Measurements and Main Results:**

Exposure to LPS temporarily increased pulmonary artery mean pressure and impeded renal function with elevated serum creatinine and acidosis compared to a control group over the 30-hour study period. Double treatment with both iNO and IV steroid tended to blunt the deterioration in renal function, although the only significant effect was on Base Excess (p = 0.045). None of the LPS + iNO + IV steroid treated animals died during the study period, whereas one animal died in each of the other LPS-infused groups.

**Conclusions:**

This study suggests that combined early therapy with iNO and IV steroid is associated with partial protection of kidney function after 30 hours of experimental LPS infusion.

## Introduction

Despite modern intensive care the hospital mortality in sepsis with multi-organ failure varies between 30–75%, with the highest mortality in patients with septic shock [1]–[4]. Inhaled nitric oxide (iNO) improves arterial oxygenation and attenuates pulmonary hypertension by selective relaxation of vascular smooth muscle cells in ventilated lung regions [5], [6]. Moreover, it has been increasingly accepted that iNO also has systemic effects [7], [8]. Neither the mechanisms, nor the extent of iNO's extrapulmonary effects, are sufficiently known. Nitric oxide (NO) in tissue has anti-inflammatory effects and inhibits the expression of cytokines, adhesion molecules, interleukins, and other inflammatory mediators [9]–[15]. INO and corticosteroid administration have both been shown to modulate the inflammatory process by, among other actions, inhibiting the activation of NF-κB, which regulates the expression of many inflammatory and immune genes [12], [14], [16].

In addition to the immunomodulatory effects, steroid administration may also have hemodynamic impact [16], [17]. A review of these mechanisms concluded that “in the absence of steroids, capillary permeability increases, vasomotor tone decreases and cardiac size as well as cardiac output decrease” [16]. In an ischemia-reperfusion model of patients undergoing knee surgery, those exposed to iNO at 80 parts per million (ppm) 30 minutes before and during tourniquet application displayed significantly reduced inflammation [7].

Da *et al* [18] hypothesized that iNO combined with intravenous (IV) hydrocortisone could have synergistic effects in the treatment of sepsis due to their similar immunomodulatory actions. In a six-hour porcine endotoxemia model, simultaneous administration of iNO at 30 ppm and IV hydorcortisone (3.5 mg/kg as a bolus dose) blunted the inflammatory response, influenced pathophysiological response in a beneficial manner, and almost preserved or restored normal histology of the lungs, liver and kidneys [18]. They also reported that pulmonary, hepatic, and renal glucocorticoid receptors were first down-regulated by endotoxin exposure and subsequently up-regulated by iNO administered during the last 3.5 hours of the model.

Though the effects observed in these studies are striking, they were only evaluated over a few hours. We considered it essential to investigate the effects of combined therapy with iNO and IV steroid over a longer period of time that would more closely reflect the clinical reality for patients developing sepsis-induced organ dysfunction. Accordingly the aim of our study was to examine whether the inflammatory response and worsened pulmonary and abdominal organ function resulting from endotoxin infusion was blunted by iNO or IV steroid alone or by a combination of iNO and IV steroid in a 30-hour porcine endotoxemia model.

## Materials and Methods

### Ethics statement

The Animal Research Ethics Committee of the Institute of Immunology and Experimental Therapy, Polish Academy of Science, Wroclaw, Poland approved the study (#7/05). Death was not used as a primary endpoint. This allowed us to humanely euthanize an animal that was unresponsive to treatment of severe circulatory collapse. Adequate anesthesia or sedation was used throughout the period of the experiment. Muscle relaxants were not used. Animals surviving to the 30-hour endpoint were euthanized with a lethal dose of IV barbiturate.

### Anesthesia and instrumentation

The experiments were conducted at the Institute of the Experimental Surgery and Biotechnology Research, Wroclaw University of Medicine. We studied 30 domestic piglets, 2 months old, and with a median body weight of 21 kg (range 15–24 kg). We have previously described this model in detail [8], [19]. The piglets were fasted overnight prior to the study. Induction of anesthesia was performed with intramuscular injection of zolazepam/tiletamin 4 mg×kg^−1^ dissolved in medetomidine 0.08 mg×kg^−1^. The trachea was intubated and the piglet was ventilated in a pressure-controlled mode using a Servo 900C ventilator (Siemens Elema-Solna, Sweden) at an inspired fraction of oxygen (FIO_2_) of 0.3 and a PEEP of 5 cm H_2_O. The inspiratory pressure was set to keep the piglets normoventilated. Anesthesia was maintained with an IV infusion of a mixture of ketamine 1.5–2.4 mg×kg^−1^×h^−1^, medetomidine (5.3–8.2 µg×kg^−1^×h^−1^), fentanyl (0.8–1.3 µg×kg^−1^×h^−1^), and midazolam (0.08–0.13 µg×kg^−1^×h^−1^). Anesthetic doses were increased during instrumentation and lowered to standard sedation doses for the remainder of the study period. Instrumentation involved the placement of a femoral arterial line (BD Careflow femoral artery catheter, Becton Dickinson, Singapore) and a transabdominal urinary bladder catheter (mini-laparatomy, Rüsch catheter, Kernen, Germany) for measurement of the urine output. We also placed, via internal jugular cutdown, a central venous catheter (CVC) (BD Careflow central venous catheter, Becton Dickinson, Singapore) and a balloon-tipped flotation pulmonary artery catheter with a thermistor (PAC) (CritiCath SP5105H TD catheter, Becton Dickinson, Singapore). Finally, a drainage tube was inserted into the abdominal cavity to evacuate any ascites fluid. The piglets were then provided a 1-hour recovery period after which baseline data ( = time zero) were registered and the initial blood specimens were drawn from the arterial catheter.

### Protocol

**Figure 1:**
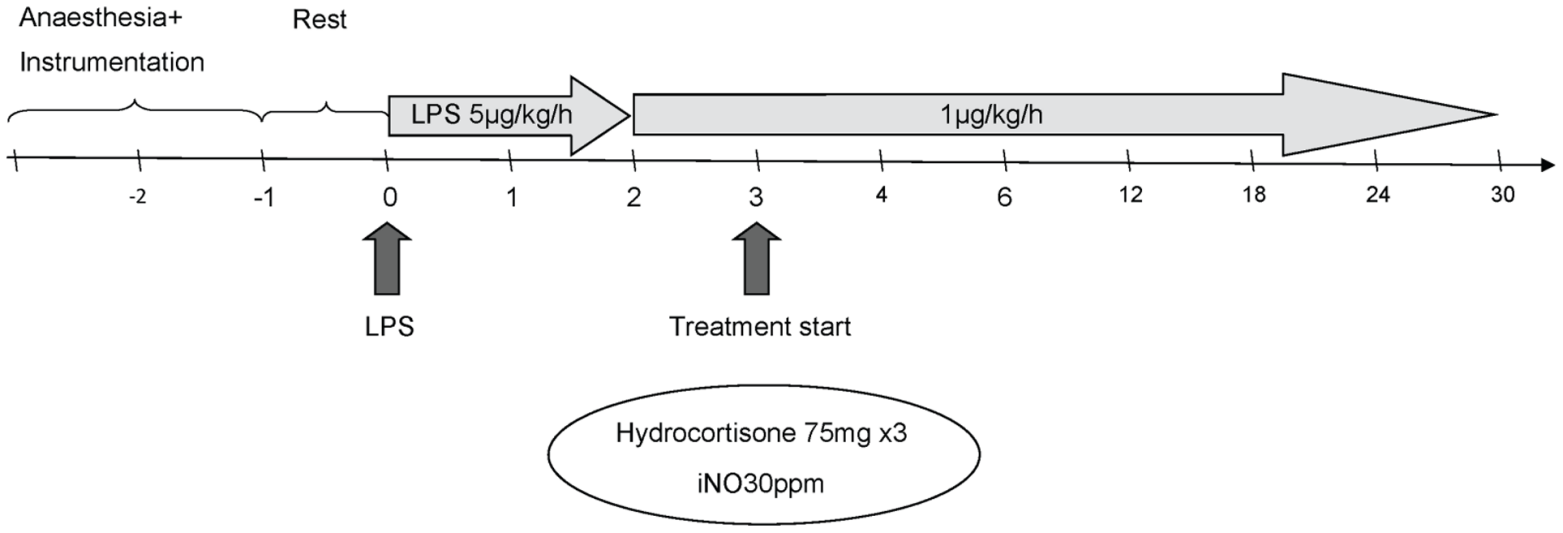
Study protocol. The scale bar depicts time in hours.

The protocol was designed to evaluate the effects of various interventions on prolonged LPS-induced organ dysfunction. A sepsis-like condition was established by continuous IV infusion of endotoxin (Lipopolysaccharide [LPS] from *Escherichia coli* [L2630-25MG], SIGMA, Gothenburg, Sweden, Chemical lot 110K41 10, mixed in sterile water  = 2 mg×ml^−1^). An initial dose of 5 µg×kg^−1^×h^−1^ was administered for two hours after baseline measurements and then lowered to 1 µg×kg^−1^×h^−1^ for the remaining 28 hours of the study period. A flowchart describing the various experimental procedures is given in Figure 1. Fluid and vasopressor support were administered per protocol (see below) to all groups.

The piglets were randomized into five groups and observed for 30 hours as follows:

1. Control: No LPS infusion, iNO, or IV steroid.
2. LPS-only: Continuous IV infusion of LPS for the whole study period (30 hours).
3. LPS + iNO: iNO at 30 ppm started after 3 hours of LPS infusion and continued until the end of the experiment.
4. LPS + IV steroid: IV hydrocortisone 75 mg started after 3 hours of LPS infusion and repeated every 8 hours thereafter.
5. LPS + iNO + IV steroid: Both continuous iNO at 30 ppm and IV hydrocortisone 75 mg every 8 hours starting 3 hours after the LPS infusion and continued until the end of the experiment.

The study was designed to include six piglets in each group and an experimental length of 30 hours, followed by sacrifice of animals surviving the protocol period. The control group was used solely to enable comparison with the LPS group.

### Administration of iNO

Inhaled nitric oxide (iNO) (Pulmonox-Messer Griesheim 800 ppm NO in 9000 nitrogen) was delivered at 30 ppm by a Pulmomix Mini (Messer Griesheim, Gumpoldskirchen, Austria) to the inspiratory limb of the ventilator as previously described by us [8].

### Monitoring and hemodynamics

The animals were monitored continuously with a three-point electrocardiogram (ECG), recordings of heart rate (HR, beats per min), mean systemic, pulmonary arterial, and central venous pressures, (MAP, MPAP and CVP, mmHg), pulse oximetry (SpO_2,_ %), fraction of inspired oxygen (FIO_2_), and end-tidal carbon dioxide concentration (EtCO_2_, kPa) (General Electric health care AS/3 Instrumentarium, OY Helsinki, Finland). Pulmonary capillary wedge pressure (PCWP, mmHg) and thermodilution cardiac output (CO, L×min^-1^) were measured every 4 hours. Urinary output was measured and the bladder emptied at 12, 24, and 30 hours. Body temperature was monitored by the PAC thermistor in order to keep the animals normothermic (37–38°C) using heating blankets or external cooling if needed. The animals were turned side to side every four hours.

Hypotension, defined as MAP less than 60 mmHg for longer than 3 minutes, was treated initially with a 300 ml bolus of lactated Ringers solution or infusion of up to 750 ml hydroxyethyl starch. If MAP remained below 60 mmHg an infusion of norepinephrine (NE) (40 µg×ml^−1^) was started. We aimed at maintaining CVP between 6 and 8 mmHg. The summed doses of NE given at 10 time-points during the study (1, 2, 3, 4, 8, 12, 16, 20, 24 and 28 hours) were reported as “total NE” for each animal.

In order to compensate for fluid loss and maintain the blood glucose level a mixture of 2.5% dextrose in saline 0.9% (Glu/NaCl 1∶1) (Braun, Melsungen, Germany) was given at a basal infusion rate of 100 mL×h^−1^. High or low blood glucose levels were not treated. Glycopyrrolate 0.2 mg×1500 mL^−1^ was added to the infusate in order to counter bradycardia. In addition, all animals received cefuroxime (GlaxoSmithKline, Solna, Sweden) 500 mg IV every 8 hours to counter accidental bacterial contamination during instrumentation.

### Laboratory specimens

Blood samples were drawn at baseline (after instrumentation and 1-hour recovery period), at regular intervals for monitoring, and after 30 hours of endotoxin exposure. Measurements included white blood cell count (WBC), interleukin 1 (IL-1), tumour necrosis factor-α (TNF_α_), creatinine, and urea. Point-of-care analysis of blood gases (ISTAT, Abbot, East Windsor, USA) included pH, PaCO_2_, PaO_2,_ and base excess (BE). Blood drawn for cytokine levels was immediately centrifuged (Hettich Zentrifugen Universal 16R, Hettich Gmb&H, Tuttlingen, Germany) for ten minutes at 4000 g×min^−1^. The supernatants were stored at −70°C until analyzed. WBC, creatinine, and urea were analyzed within one hour.

### Interventions

The protocol allowed for certain pre-specified actions to mimic clinical treatment. These included fluid boluses and IV norepinephrine if MAP remained below 60 mm Hg for more than 3 minutes, increased inspiratory pressure and FIO_2_ to maintain normocarbia and avoid hypoxemia, and endotracheal suction for secretion clearance. Metabolic acidosis was not treated.

### Statistics

Differences within groups between baseline and 30 hours were tested using the exact Wilcoxon signed rank test. Treatment effect, measured as the difference from baseline to 30 hours, was compared between the three treatment groups (iNO, IV steroid and iNO + IV steroid) and the LPS-only group using the exact Wilcoxon-Mann-Whitney test. Given the complexity of the study with experiments lasting for 35 hours or more (including setup and termination) we chose to limit group size to 6 animals to make the study feasible. For animals that died prior to the full study period we used the last antemortem data for analysis, provided that the animal had lived at least 20 hours. This was the case in 2 of the 3 deaths. Multiplicity adjustments were done using the Bonferroni-Holm procedure for the between-groups comparisons. A much larger number of animals would have been required for comparisons and multiplicity adjustments within groups, and was not the main focus of the study. Two-sided tests were used. A *p*-value of <0.05 was taken to indicate a statistically significant difference.

## Results

### General findings

Before LPS infusion there were no baseline differences between study groups in respiratory or circulatory variables or renal function, although the double treatment group tended to have higher PaO_2_/FIO_2_ than the LPS-only group (Tables 1–3). No significant effects were seen over time or between groups on HR, CVP, or PCWP (data not shown). The total amount of anesthetic and fluids given during the study period was not significantly different between all groups receiving LPS. No animals died in the control group or the LPS + iNO + IV steroid (“double-treatment”) group, and there was 1 death each in the remaining three LPS-exposed groups. One animal died of respiratory failure (pulmonary edema) at 6 hours, one animal died of sudden arrhythmia late in the study period. A third animal was euthanized per protocol (close to the end) after having developed severe and unresponsive hypotension.

**Table 1:** Respiratory variables.

	Group	Baseline	30 h	P^1^	P^2^	P^3^ (P adj)
**Peak Inspiratory Pressure (cmH_2_O)**	Control	16; 13–19	19; 18–22	0.06	Ref.	
	LPS-only	15; 15–24	28; 23–35	0.05	0.005	
	LPS + iNO	17; 16–19	24; 22–40	0.03		1.0 (1.0)
	LPS + IV steroid	18; 13–26	27; 17–40	0.09		0.86 (1.0)
	LPS + iNO + IV steroid	17; 15–20	20; 14–37	0.16		0.64 (1.0)
**PaO_2_/FIO_2_ (kPa)**	Control	48; 36–77	45; 35–98	0.68	Ref.	
	LPS-only	52; 45–57	48; 13–57	0.36	0.78	
	LPS + iNO	64; 51–92	45; 28–79	0.16		1.00 (1.0)
	LPS + IV steroid	54; 43–112	44; 19–76	0.03		0.43 (1.0)
	LPS + iNO + IV steroid	66; 59–98	56; 44–90	0.22		0.93 (1.0)
**PaCO_2_ (kPa)**	Control	5.79; 4.45–7.01	4.48; 3.37–6.49	0.07	Ref.	
	LPS-only	5.67; 4.70–6.93	5.53; 4.60–5.82	0.44	0.41	
	LPS + iNO	5.73; 3.72–6.88	4.70; 4.17–6.55	0.44		0.66 (1.0)
	LPS + IV steroid	5.22; 4.48–7.39	5.28; 4.28–9.30	1.00		0.54 (1.0)
	LPS + iNO + IV steroid	5.17; 4.75–6.67	5.09; 4.67–7.43	0.84		0.57 (1.0)

**Statistics**: Values are shown as median and range.

1 Within group from baseline to 30 hours.

2 Comparison of distribution of differences between LPS-only and control.

3 Comparison of distribution of differences against LPS-only (Bonferroni-Holm adjusted p-values within parenthesis).

**Table 2:** Circulatory variables.

	Group	Baseline	30 h	P^1^	P^2^	P^3^ (P adj)
**Cardiac output (L/min)**	Control	2.4; 2.2–4.4	2.1; 1.2–.6	0.06	Ref.	
	LPS-only	2.0; 1.3–2.3	1.4; 1.1–3.7	0.81	0.24	
	LPS + iNO	2.5; 1.9–4.4	3.5; 1.4–4.5	0.62		0.73 (0.92)
	LPS + IV steroid	2.4; 1.9–4.4	3.5; 1.4–4.5	0.12		0.46 (0.92)
	LPS + iNO + IV steroid	2.7; 2.1–2.8	1.9; 1.3–2.8	0.09		0.19 (0.58)
**MAP (mmHg)**	Control	101; 92–124	84; 63–96	0.15	Ref	
	LPS-only	93; 79–151	110; 87–138	0.81	0.64	
	LPS + iNO	91; 52–103	82; 63–138	1.00		0.69 (1.0)
	LPS + IV steroid	95; 75–103	82; 63–138	1.00		1.00 (1.0)
	LPS + iNO + IV steroid	92; 63–124	100; 72–129	0.78		0.70 (1.0)
**MPAP (mmHg)**	Control	15; 12–19	17; 12–18	0.85	Ref.	
	LPS-only	15; 11–21	25; 17–34	0.12	0.01	
	LPS + iNO	12; 7–19	15; 12–37	0.06		0.56 (0.56)
	LPS + IV steroid	15; 13–23	32; 15–41	0.06		0.08 (0.24)
	LPS + iNO + IV steroid	13; 11–21	14; 8–38	0.50		0.26 (0.51)
**Total NE adm. (µg/kg)**	Control	0.0; 0–0				
	LPS-only	162; 9–1169		0.001		
	LPS + iNO	64; 0–1071				0.63 (0.63)
	LPS + IV steroid	37; 0.0–72				0.20 (0.40)
	LPS + iNO + IV steroid	7; 0–45				0.04 (0.11)

**Statistics**: Values are shown as median and range.

1 Within group from baseline to 30 hours.

2 Comparison of distribution of differences between LPS-only and control.

3 Comparison of distribution of differences against LPS-only (Bonferroni-Holm adjusted p-values within parenthesis).

**Table 3:** Kidney function.

	Group	Baseline	30 h	P^1^	P^2^	P^3^ (P adj)
**pH**	Control	7.43; 7.42–7.54	7.46; 7.38–7.55	1.00	Ref.	
	LPS-only	7.46; 7.45–7.52	7.30; 7.25–7.32	0.06	0.004	
	LPS + iNO	7.46; 7.39–7.54	7.34; 7.21–7.42	0.03		0.33 (0.61)
	LPS + IV steroid	7.47; 7.33–7.52	7.33; 7.17–7.40	0.03		0.31 (0.61)
	LPS + iNO + IV steroid	7.47; 7.40–7.50	7.41; 7.27–7.54	0.56		0.18 (0.54)
**BE (mEq/L)**	Control	+6.0; +1.0–+8.0	+1.5; −9.0–+7.0	0.06	Ref.	
	LPS-only	+6.0; +4.0–+8.0	−8.0; −8.0–−5.0	0.06	0.004	
	LPS + iNO	+6.5; +2.0–+9.0	−6.5; −11.0–+4.0	0.03		0.83 (0.83)
	LPS + IV steroid	+4.0; +3.0–+7.0	−6.0; −9.0–+1.0	0.03		0.017(0.045)
	LPS + iNO + IV steroid	+4.0; +0.0–+6.0	−0.5; −6.0–+8.0	0.31		0.015(0.045)
**Urea (mmol/L)**	Control	4.67; 2.17–5.33	2.34; 1.67–3.00	0.03	Ref.	
	LPS-only	4.34; 3.50–6.67	11.00; 2.33–17.67	0.31	0.02	
	LPS + iNO	3.83; 3.50–6.33	8.92; 5.83–16.17	0.03		0.93 (1.0)
	LPS + IV steroid	3.67; 1.50–6.33	9.67; 4.83–27.17	0.03		0.93 (1.0)
	LPS + iNO + IV steroid	3.75; 2.33–7.60	8.17; 3.33–13.67	0.22		0.79 (1.0)
**Urine output 12–30 hours, (mL)**	Control	1040; 200–1500			Ref.	
	LPS-only	580; 400–1160			0.58	
	LPS + iNO	1058; 0–1790				0.66 (1.0)
	LPS + IV steroid	716; 150–2095				0.47 (1.0)
	LPS + iNO + IV steroid	1025; 400–2500				0.26 (0.79)

**Statistics**: Values are shown as median and range.

1 Within group from baseline to 30 hours (urine output: no baseline value, volume collected over time, from 12 to 30 hours).

2 Comparison of distribution of differences between LPS-only and control.

3 Comparison of distribution of differences versus LPS-only (Bonferroni-Holm adjusted p-values within parenthesis).

### LPS group

**Figure 2:**
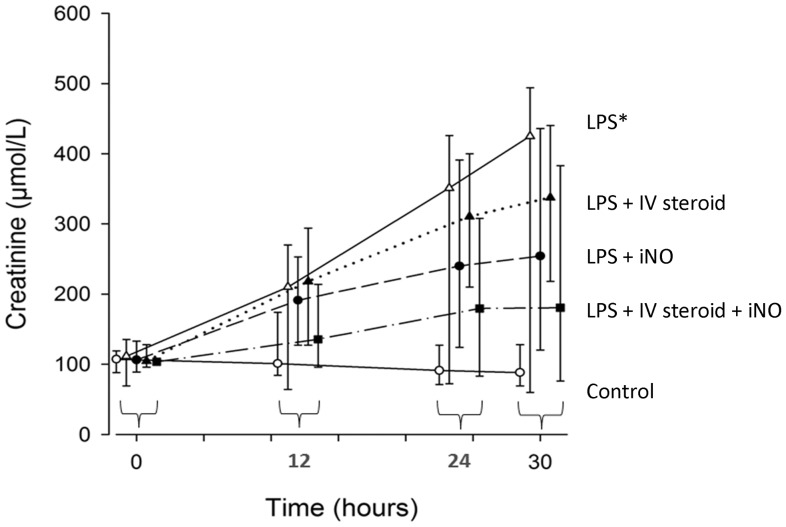
Changes in serum creatinine levels over time, unit  = µmol/L. LPS  =  lipopolysaccharide, IV steroid  =  intravenous hydrocortisone, iNO  =  inhaled nitric oxide. Values are shown as median and range. Data have been collected at 0, 12, 24 and 30(*: p = 0.02). No significances between treatment groups (IV steroid, iNO, IV steroid + iNO) and LPS alone were noted.

LPS exposure over 30 hours was associated with increased peak inspiratory pressure compared to baseline (p = 0.05) and compared to controls (LPS-only vs control, p = 0.005; Table 1). MPAP also increased compared to control (p = 0.01; Table 2). Total norepinephrine dose was also higher in this group (LPS-only vs control, p = 0.001; Table 2). pH and BE worsened compared to controls (p = 0.004 for both; Table 3) as did urea (p = 0.02; Table 3) and serum creatinine (p = 0.02; Fig. 2). Inflammatory mediator concentrations (WBC, IL-1, TNF-α) were increased at 30 hours but with borderline significances (p = 0.06 for all three; Table 4). TNF-α was also increased in LPS-only animals compared to control (p = 0,02; Table 4).

**Table 4:** Inflammatory mediators.

	Group	Baseline	30 h	P^1^	P^2^	P^3^ (P adj)
**WBC**	Control	11.9; 4.4–15.3	11.9; 8.5–14.7	0.44	Ref.	
	LPS-only	7.5; 4.5–13.3	18.3; 7.6–21.8	0.06	0.25	
	LPS + iNO	8.8; 4.0–20.1	34.5; 3.9–43.9	0.50		1.00 (1.0)
	LPS + IV steroid	7.4; 3.7–16.5	21.4; 8.5–65.5	0.06		0.15 (0.45)
	LPS + iNO + IV steroid	11.8; 5.8–18.8	17.5; 8.4–40.1	0.16		0.97 (1.0)
**IL-1**	Control	17.5; 9.6–84	18.4; 4–48	1.00	Ref.	
	LPS-only	30.7; 4–68	117; 9.6–186	0.06	0.06	
	LPS + iNO	26.2; 8–72	75; 42–116	0.18		0.41 (1.0)
	LPS + IV steroid	44.1; 8–72	75; 41.8–116	0.25		0.90 (1.0)
	LPS + iNO + IV steroid	18.9; 2. 70	50; 14–130	0.12		0.84 (1.0)
**TNF-α**	Control	88; 53–130	97; 66–117	0.81	Ref.	
	LPS-only	77; 63–110	599; 154–1077	0.06	0.02	
	LPS + iNO	81; 62–130	521; 106–1243	0.12		0.73 (1.0)
	LPS + IV steroid	72; 62–99	429; 277–679	0.12		0.91 (1.0)
	LPS + iNO + IV steroid	77; 54–203	243; 153–443	0.06		0.55 (1.0)

**Statistics**: Values are expressed as median and range.

WBC  =  White blood cell count (x10^9^/L); IL-1 =  Interleukin 1(pg/ml); TNF-α =  Tumor Necrosis Factor-α (pg/ml); LPS =  Lipopolysaccharide; iNO =  Inhaled nitric oxide.

1 within group from baseline to 30 hours.

2 Comparison of distribution of differences between LPS-only and control.

3 Comparison of distribution of differences against LPS-only (Bonferroni-Holm adjusted p-values within parenthesis).

### Treatment groups

No significant effects of iNO, IV steroid or a combination of both were seen in respiratory or circulatory variables (Table 1, 2), but the double treatment group (iNO + IV steroid) tended to receive less norepinephrine than the LPS-only group (p = 0.12; Table 2). BE decreased less in the IV steroid only and iNO + IV steroid groups than in the LPS-only group (p = 0.045 for both after Bonferroni-Holm correction; Table 3). Increases were seen in urea, and serum creatinine, and decrease in pH, in all three treatment groups (Fig. 2, Table 3). The amount of urine collected over the latter 12 to 30 hours varied considerably within groups, without a clear or statistically significant pattern (Table 3). The concentration of inflammatory mediators at 30 hours in the three treatment groups did not differ from the LPS-only group (Table 4).

## Discussion

In this study we found no significant effect of combination therapy with inhaled nitric oxide and intravenous steroid in an experimental porcine model of extended challenge with endotoxin, although a tendency to a protection of kidney function was seen.

Based on our previous experience with prolonged experimental studies in a porcine model [8], [19], this protocol involved 30 hours of endotoxin exposure using high-dose E. coli lipopolysaccharide (LPS) infusion, which resulted in multiple organ dysfunction after 12–24 hours. This study was intended to mimic the clinical setting, with an initial endotoxin insult and then resuscitative interventions initiated in real time over a prolonged study period.

Early in the experiment (4–6 hours) transient intense pulmonary hypertension was evident in all animals exposed to LPS. We did not focus on these early events as they have been described in detail in several earlier studies [18], [20], [21]. A majority of animals developed signs of impaired organ function and deaths occurred in all groups exposed to LPS except in the double-treated group.

When initially going over our data, it became clear to us that our interventions to some extent blunted the monitored pathophysiological response. Our protocol allowed for various interventions, e.g. against hypotension, hypovolemia, hypoxemia and hypercarbia which we believe made most animals survive the study period of 30 hours. On the other hand, these interventions reduced the possibility to detect statistically significant differences in pathophysiology between the study groups.

The animals treated with the combination of iNO + IV steroid tended to be more stable and less sick during the protocol period of 30 hours of LPS-infusion, as compared to LPS-only animals. Several animals required vasopressor support in addition to increased crystalloid administration in order to maintain MAP >60 mmHg. Although not statistically significant, this seemed to occur less frequently in the LPS + IV steroid and LPS + iNO + IV steroid groups. In general, we seldom encountered severe respiratory problems in the treatment groups, with only few animals requring FIO_2_>0.50. We noted an increased peak inspiratory pressure (PIP) in several LPS-exposed animals, again with a tendency of less rise in the double treatment (Table 1). A rise in airway pressure could be a sign of an inflammatory response in lung tissue and is a hallmark of acute lung injury [22]. Acute kidney injury (AKI), signified by polyuria or oliguria plus elevated creatinine, urea, and decreasing Base Excess, was noted in many animals exposed to LPS. Clinical studies have shown that the presence of AKI as ‘risk’ increases hospital mortality from 8.4% to 20.9%, and when ‘injury’ is present, to 45.6% [23], [24].

Recent data from Hållström *et al* suggest interspecies differences in the response to iNO during experimental endotoxemia [25], [26]. However the much smaller endotoxin dose used in these studies on healthy human volunteers compared to our experimental animals should also be kept in mind. In addition, the administration of endotoxin is not identical to a septic state, in which also live bacteria and possibly release of toxins other than LPS complicate the biochemistry and pathophysiology. It can also be discussed which array of cytokines and markers to follow, during the exposure to endotoxin. In a recent review by Pierrakos and Vincent [27], a total of 178 biomarkers were discussed and referenced. It was concluded that no one special marker has yet been proven in experimental and clinical studies to be of greater relevance and specificity than the others.

Furthermore, the present piglet study and other work to date have not discerned which doses of iNO and IV hydrocortisone are optimal for clinical effect, nor have they defined whether a pre-emptive treatment strategy would yield a stronger effect. In a recent review, Rivers et al [28] argue for start of immunotherapeutic intervention early, before the cytokine storm has blown over. Finally, we do not yet know the exact molecular mechanism for the modifying effect, if any, of a combined treatment with iNO and IV steroids, during an LPS infusion. Modified expression of the glucocorticoid receptor remains a possibility [18]. An interesting additional idea would be a reduced expression of Toll-like receptors, in similarity with what was preliminarily reported earlier this year from a study focusing on Toll-like receptor 4 (TLR 4) during ischemia-reperfusion injury in a piglet model [29].

## Conclusions

We have studied sedated and mechanically ventilated piglets during a prolonged (30 hour) LPS infusion examining clinically meaningful endpoints while intervening with iNO and IV steroid separately and in combination. Although a tendency of organ protection was noted by the combination of iNO and IV steroid, in particular of the kidney, no clearly significant differences were seen. A more focused study in a larger material may reveal significant differences.
